# How is personality related to research performance? The mediating effect of research engagement

**DOI:** 10.3389/fpsyg.2023.1257166

**Published:** 2024-01-10

**Authors:** Rui Feng, Yunhui Xie, Junjie Wu

**Affiliations:** ^1^School of Economics and Management, Beihang University, Beijing, China; ^2^Human Resources Department, Beihang University, Beijing, China; ^3^Key Laboratory of Data Intelligence and Management (Beihang University), Ministry of Industry and Information Technology, Beijing, China

**Keywords:** personality, Big-Five theory, research engagement, research performance, university

## Abstract

Faculty members' research performance holds great significance for the development of a university. The primary objective of this study is to examine the influences of researchers' personalities on their research performance within universities, as well as the mediating role of research engagement in this relationship. The study encompassed 189 faculty members from a university and employed descriptive statistics, correlation analysis, measurement, and structural equation modeling as the analytical procedures. The results obtained from structural equation modeling reveal significant effects of faculty members' personalities on their objective research performance rather than self-reported performance. Specifically, conscientiousness and openness to experience exhibit a positive correlation with research performance. On the contrary, the neuroticism and social attributes of personality (the integration of extraversion and agreeableness) exhibit a negative correlation with research performance. Furthermore, research engagement mediates the effects of openness to experience and neuroticism on research performance. This study carries significant implications for the training and recruitment selection of faculty members in universities and enhances our understanding of how different personalities lead to a variance in research engagement and performance.

## 1 Introduction

Research performance refers to the achievement and efficiency gained by researchers who engage in scientific research activities and other related works (Zhang et al., 2014; Guo et al., 2017). It serves as a reflection of faculty members' creativity, efficiency, and productivity in a university. It also promotes the university's educational quality by improving the instructors' expertise, thereby increasing students' knowledge and finally boosting the university's standing (Horodnic and Zait, 2015). Therefore, the significance of research performance in developing a university cannot be overstated. Hence, undertaking an exploration of the factors and mechanisms that influence faculty members' research performance holds considerable significance.

Several studies have explored the antecedents of research performance among faculty members in universities. However, empirical research focusing on research performance remains limited compared to the vast body of empirical research on work performance. Previous researchers have identified some key factors that affect research performance, such as gender differences (Bentley, 2011; Nielsen, 2015; Zhang et al., 2020), academic pressure (Wang et al., 2013; Zhang et al., 2014), job involvement (Keller, 1997), work engagement (Kim et al., 2012), motivation (Horodnic and Zait, 2015), international academic collaboration (Zhang et al., 2020), bullying (Meriläinen et al., 2019), self-regulation (Diotaiuti et al., 2021), and scientists' attitudes toward gender equality and work-life balance (Ko et al., 2020). However, only a few studies have investigated the relationship between personality and research performance among researchers (Sherry et al., 2010; Guo et al., 2017; Kim and Choi, 2017; Djupe et al., 2020; Lindahl, 2023).

Personality affects how individuals perceive and deal with the environment in a consistent way (Grosul and Feist, 2014). People with different personality traits may exhibit distinct emotions, interpretations, and actions, even when confronted with identical situations and challenges. The Big Five personality traits include extraversion, agreeableness, conscientiousness, neuroticism, and openness to experience. Because the Big Five personality traits serve as a meaningful taxonomy for classifying personality, they have been widely used as predictors of job performance (Barrick and Mount, 1991; Hurtz and Donovan, 2000; Barrick et al., 2001; Poropat, 2009; Grosul and Feist, 2014; Lea et al., 2020; Serrano et al., 2022). The effects of the Big Five personality traits have been verified on student academic performance (Poropat, 2009; Serrano et al., 2022), ERP system learning performance (Lea et al., 2020), work engagement and job satisfaction (Woods and Sofat, 2013; Akhtar et al., 2015; Li et al., 2015, 2021; Mroz and Kaleta, 2016), and so on.

While the impact of personality on work performance has been extensively studied, there is a surprising dearth of empirical research exploring the relationship between the Big Five personality traits of faculty members and research performance in academic settings. Only a limited number of studies have investigated the relationship between personality and research productivity (Mallinckrodt and Gelso, 2002; Sherry et al., 2010; Guo et al., 2017; Kim and Choi, 2017; Djupe et al., 2020; Lindahl, 2023). For example, Djupe et al. (2020) found that faculty members' conscientiousness and openness to experience predict high productivity in political science, and these two personality traits have compensatory effects such that each matters most in the absence of the other. Lindahl (2023) found that conscientiousness is positively related to research productivity among doctoral students during their doctoral studies. Sherry et al. (2010) explored the relationship between self-oriented perfectionism, conscientiousness, socially prescribed perfectionism, neuroticism, and research productivity in psychology professors and found that self-oriented perfectionism is negatively related to research productivity in psychology professors. However, fewer studies have examined the mediating mechanism of personality's impacts on research performance. Research engagement may serve as a pivotal mediator in understanding the relationship between personality traits and research performance, given that work engagement typically acts as a mediator between antecedents (such as job resources and personal resources) and consequences (such as performance; Kim et al., 2012; Schaufeli and Taris, 2014; Mudrak et al., 2017; Schaufeli, 2017; Han et al., 2019). Specifically, personality exerts an influence on work engagement. For example, individuals with a conscientious disposition are more prone to experiencing high levels of work engagement (e.g., Rossier et al., 2012; Scheepers et al., 2016; Zhou and Tang, 2022). Higher neuroticism leads to lower work engagement (Perera et al., 2018; Li et al., 2021) and lower career engagement (McIlveen and Perera, 2016), as neuroticism may cause a greater diversion of attention from work and career. Openness to experience is positively correlated with work engagement for teachers (Zaidi et al., 2013; Li et al., 2021), as it predicts vigor, dedication, and absorption (Douglas et al., 2016). The positive effect of extraversion and agreeableness on work engagement has also been demonstrated by scholars (Zaidi et al., 2013; Perez-Fuentes et al., 2019; Zhou and Tang, 2022). Furthermore, research engagement exhibits a significant correlation with research performance (Guo et al., 2017).

Therefore, this study focuses on how faculty members' personality traits affect their research performance and how this relationship is mediated by research engagement.

## 2 Theory and hypotheses

### 2.1 “Big Five” personality traits and research performance

Personality is defined as “an individual's characteristic patterns of thought, emotion, and behavior, together with the psychological mechanisms—hidden or not—behind those patterns” (Funder, 2001, p. 2). Scientists have attempted to identify the basic dimensions of personality for decades (Goldberg, 1990; McCrae and John, 1992). Until the 1990's, the Big Five or Five-Factor Model (FFM) was the best-established model to describe personality (Herr et al., 2021). The model comprises five fundamental features: extraversion, agreeableness, conscientiousness, neuroticism, and openness to experience (Goldberg, 1990; McCrae and John, 1992). It has been widely used and accepted in personality research to predict behavior and outcome due to its robust, replicable, convergent, and discriminant validity (Goldberg, 1990; Barrick and Mount, 1991; McCrae and John, 1992; Barrick et al., 2001; Grosul and Feist, 2014).

The study of Big Five personality traits and their impact on performance has garnered significant attention from researchers in various fields, including psychology, organizational behavior, and education. Earlier research has found that some personality dimensions of the Big Five are related to the performance of all jobs, whereas other personality dimensions are only valid for a few jobs and some criterion types (Barrick and Mount, 1991; Hurtz and Donovan, 2000). Hurtz and Donovan (2000) concluded that personality traits other than conscientiousness played almost equally important roles in some occupations and criteria. Most researchers appear to be of the consensus that conscientiousness is generally a valid positive predictor of job performance, whereas neuroticism plays a negative role (Barrick et al., 2001; Kim et al., 2009; Poropat, 2009; Neal et al., 2012; Li et al., 2021; Serrano et al., 2022). Barrick et al. (2001) summarized prior works and found that conscientiousness was a valid predictor for all occupations studied, and emotional stability (neuroticism) was a generalizable predictor. However, the other three personality traits did not exhibit significant predictive power in overall work performance (Barrick et al., 2001).

Recent literature indicates that the five dimensions of personality have distinct impacts on research performance. For instance, conscientiousness and openness are associated with students' academic performance (Poropat, 2009; Serrano et al., 2022). Grosul and Feist determined that openness to experience and neuroticism could explain the variance in scientific creativity (Grosul and Feist, 2014). Li et al. (2021) found that higher openness, agreeableness, conscientiousness, and lower neuroticism positively correlate with young teachers' work engagement and job satisfaction. Additionally, researchers' conscientiousness predicts high research productivity (Djupe et al., 2020; Lindahl, 2023), and openness to experience predicts high research productivity (Djupe et al., 2020).

#### 2.1.1 Conscientiousness and research performance

Conscientiousness reflects an individual's responsibility and reliability; highly conscientious people are responsible, hardworking, well-organized, and persistent (Woods and Sofat, 2013; Mroz and Kaleta, 2016; Herr et al., 2021; Li et al., 2021). Based on these assumptions, we anticipate a positive association between conscientiousness and research performance because it assesses personal characteristics, such as being responsible, hardworking, and persistent, which are crucial attributes for achieving success in research endeavors. Therefore, the following hypothesis is proposed.

**H1a:** Conscientiousness is positively related to research performance.

#### 2.1.2 Neuroticism and research performance

Neuroticism describes an individual's emotional regulation in the face of negative experiences; Those with high levels of neuroticism tend to display impulsive reactions, feelings of depression, anxiety, and anger when confronted with adverse circumstances (Woods and Sofat, 2013; Mroz and Kaleta, 2016; Herr et al., 2021; Li et al., 2021). It is anticipated that neuroticism exerts a detrimental influence on research performance, as it engenders discouragement, self-doubt, depressive tendencies, and even abandonment, impeding progress amidst the challenges, obstacles, and setbacks encountered throughout the research process. Thus, the following hypothesis is proposed.

**H1b:** Neuroticism is negatively related to research performance.

#### 2.1.3 Openness to experience and research performance

Openness to experience represents an individual's curiosity and creativity; people with high openness are usually curious, imaginative, and eager for new things and challenges (Woods and Sofat, 2013; Mroz and Kaleta, 2016; Herr et al., 2021; Li et al., 2021). It is anticipated that openness to experience will exert a positive influence on research performance, owing to its inherent attributes of curiosity, originality, and expansive thinking, all of which are paramount for advancing research endeavors. Therefore, the following hypothesis is proposed.

**H1c:** Openness to experience is positively related to research performance.

#### 2.1.4 Social attributes of personality and research performance

Some researchers have made attempts to transition from a variable-centered approach to personality to a person-centered approach to personality (Herr et al., 2021). Herr et al. (2021) employed a person-centered approach and clustered participants into five personality types: ordinary, resilient, strained, overcontrolled, and undercontrolled, according to their Big Five personality traits. Similarly, in this study, two dimensions of the Big Five personality traits, extraversion, and agreeableness, are amalgamated into a single personality type named “the social attributes of personality,” representing the individuals' interpersonal and communication characteristics in social activities. Extraversion refers to an individual's characteristics in social activities; highly extroverted people are sociable, talkative, and active. Agreeableness refers to an individual's friendly attitude and nature; people with high agreeableness are full of kindness, warmth, empathy, trust, and cooperativeness. Feeling other people's emotions is essential to guide people's actions (Diotaiuti et al., 2022). Based on the aforementioned considerations, faculty members with high social attributes of personality tend to prefer social interactions rather than solitary research endeavors. However, such social activities may divert their focus and diminish their research engagement. As a result, we expected that the social attributes of personality may negatively affect research performance.

**H1d:** The social attributes of personality are negatively related to research performance.

### 2.2 Work engagement as a mediator

Work engagement has been proposed as a positive, fulfilling, work-related state of mind characterized by vigor, dedication, and absorption (Schaufeli and Bakker, 2004). Specifically, vigor refers to high levels of energy, willingness to invest effort in work, and persistence when encountering difficulties. A sense of pride, inspiration, enthusiasm, and challenge characterizes dedication. Absorption refers to being completely engrossed in one's work (Schaufeli et al., 2002).

Work engagement was incorporated into the job demand-resource model (JD-R model) as a mediator between resources and consequences by Schaufeli and Bakker (2004). Job demands were defined as physical, social, or organizational aspects of the job that require sustained physical or mental effort, such as work overload, work pressure, role conflict, and job insecurity, while job resources refer to a positive value, such as social support and autonomy (Demerouti et al., 2001; Bakker et al., 2005; Schaufeli and Taris, 2014). The JD-R model has two processes: a health impairment process and a motivational process. Work engagement plays an important role in the motivational process as the mediator in the relationship between job resources and positive outcomes (e.g., work performance; Schaufeli and Taris, 2014; Schaufeli, 2017).

#### 2.2.1 Work engagement and research performance

Earlier empirical studies have indicated the findings mentioned above. For example, work engagement leads to a positive work attitude and low absenteeism (Schaufeli et al., 2009) and positively affects organizational commitment (Hakanen et al., 2008), job satisfaction (Li et al., 2015; Han et al., 2019), and work performance (Keller, 1997; Kim et al., 2012; Wang et al., 2013; Zhang et al., 2014; Guo et al., 2017). Kim et al. (2012) summarized 20 empirical studies on the relationship between work engagement and work performance, referring to firefighters, part-time workers, white-collar workers, nurses, hotels, and other industries from China, the United States, Spain, Portugal, Greece, etc. Among the 20 empirical studies, they found that 11 studies reported a direct or indirect relationship between work engagement and performance, and seven reported work engagement mediating between other constructs and performance (Kim et al., 2012). Teachers' scientific research performance is also highly correlated with research engagement (Guo et al., 2017). According to the above evidence, the following hypothesis is proposed.

**H2:** Research engagement is positively related to research performance.

#### 2.2.2 Work engagement as a mediator between personality and research performance

Work engagement was found to have a mediating role in the relationship between other constructs and performance, such as fluctuations in colleague support, daily variations in job and personal resources, perceived organizational support, core self-evaluations, transformational leadership of supervisors and self-efficacy, procedural justice and so on (Kim et al., 2012). Thus, work engagement might mediate the relationship between personality traits and research performance.

Conscientious individuals are hardworking and have strong internal drives and an achievement orientation, which implies the capacity for vigor, dedication, and absorption at work (Macey and Schneider, 2008; Akhtar et al., 2015). In addition, they are less likely to be affected by external work interference, such as family-related disruptions (Halbesleben et al., 2009). Thus, highly conscientious individuals are more likely to be engaged in work (Rossier et al., 2012; Scheepers et al., 2016).

Moreover, other empirical studies have verified the positive effect of conscientiousness on work engagement (Halbesleben et al., 2009; Kim et al., 2009; Inceoglu and Warr, 2011; Joseph et al., 2011; Rossier et al., 2012; Zaidi et al., 2013; Akhtar et al., 2015; Scheepers et al., 2016; Perez-Fuentes et al., 2019; Li et al., 2021; Zhou and Tang, 2022). Consequently, the following hypothesis is proposed.

**H3a:** Research engagement mediates the positive effect of conscientiousness on research performance.

Individuals with neurotic personalities have stronger reactions to negative emotions such as fear, depression, anger, and frustration, which may have a negative effect on the capacity for vigor, dedication, and absorption at work. Higher neuroticism may cause greater diversion of attention or even withdrawal from work, leading to lower work engagement (Perera et al., 2018; Li et al., 2021). Langelaan et al. (2006) showed that work engagement is characterized by low neuroticism combined with high extraversion. Akhtar et al. (2015) found that neuroticism played a non-significant role in predicting work engagement. In addition, Douglas et al. (2016) found that withdrawal, one aspect of neuroticism, was negatively correlated with work engagement, and volatility, the other aspect of neuroticism was unrelated to work engagement. McIlveen and Perera (2016) also found that teachers' neuroticism could predict lower career engagement. By scrutinizing the literature, most believe that neuroticism can be viewed as a negative predictor of work engagement (Mostert and Rothmann, 2006; Kim et al., 2009; Joseph et al., 2011; Woods and Sofat, 2013; Zaidi et al., 2013; Perez-Fuentes et al., 2019; Zhou and Tang, 2022). Thus, we expect the following hypothesis:

**H3b:** Research engagement mediates the negative effect of neuroticism on research performance.

Among the five personality traits in the Big Five model, conscientiousness and neuroticism are considered the most reliable and second-best predictors of performance, respectively. The other three factors receive less attention and often produce inconsistent conclusions (Doo et al., 2020). For example, openness to experience predicts training proficiency but not job proficiency (Kim et al., 2009; Akhtar et al., 2015). Thus, it is not expected to be related to work engagement (Kim et al., 2009; Griffin and Hesketh, 2010). Douglas et al. (2016), who investigated two aspects of openness to experience, found that openness predicted vigor and dedication, while intellect predicted absorption. However, openness to experience was found to be positively correlated with work engagement for primary, middle, and high school teachers (Li et al., 2021), university teachers (Zaidi et al., 2013), massive open online course instructors (Doo et al., 2020), and nursing professionals (Perez-Fuentes et al., 2019). Individuals with high openness to experience tend to be curious and imaginative, which is helpful for innovation behavior. Thus, it is expected that openness to experience is positively associated with research engagement and research performance in the academic area.

**H3c:** The positive effect of openness to experience on research performance is mediated by research engagement.

In this study, extraversion and agreeableness were clustered into one personality factor defined as the social attributes of personality, which stands for the individuals' interpersonal and communication skills in social activities. Although some studies have demonstrated the positive effect of extraversion and agreeableness on work engagement (Zaidi et al., 2013; Perez-Fuentes et al., 2019; Zhou and Tang, 2022), others have argued that extraversion (Kim et al., 2009) and agreeableness (Kim et al., 2009; Woods and Sofat, 2013; Akhtar et al., 2015) do not show any significant relationships with work engagement. One potential reason for the inconsistency may be the different characteristics of different occupations of research objects. Moreover, a significant difference between teaching and research for university teachers has also been observed. Considering the unique nature of research work, teachers prefer to be alone and in a quiet environment rather than a sociable and active environment; they enjoy the experience of concentrating on scientific research without too many external distractions. Therefore, it is expected that low social attributes of personality relate to high research engagement and performance levels.

**H3d:** Research engagement mediates the negative effect of social attributes of personality on research performance.

**Figure 1:**
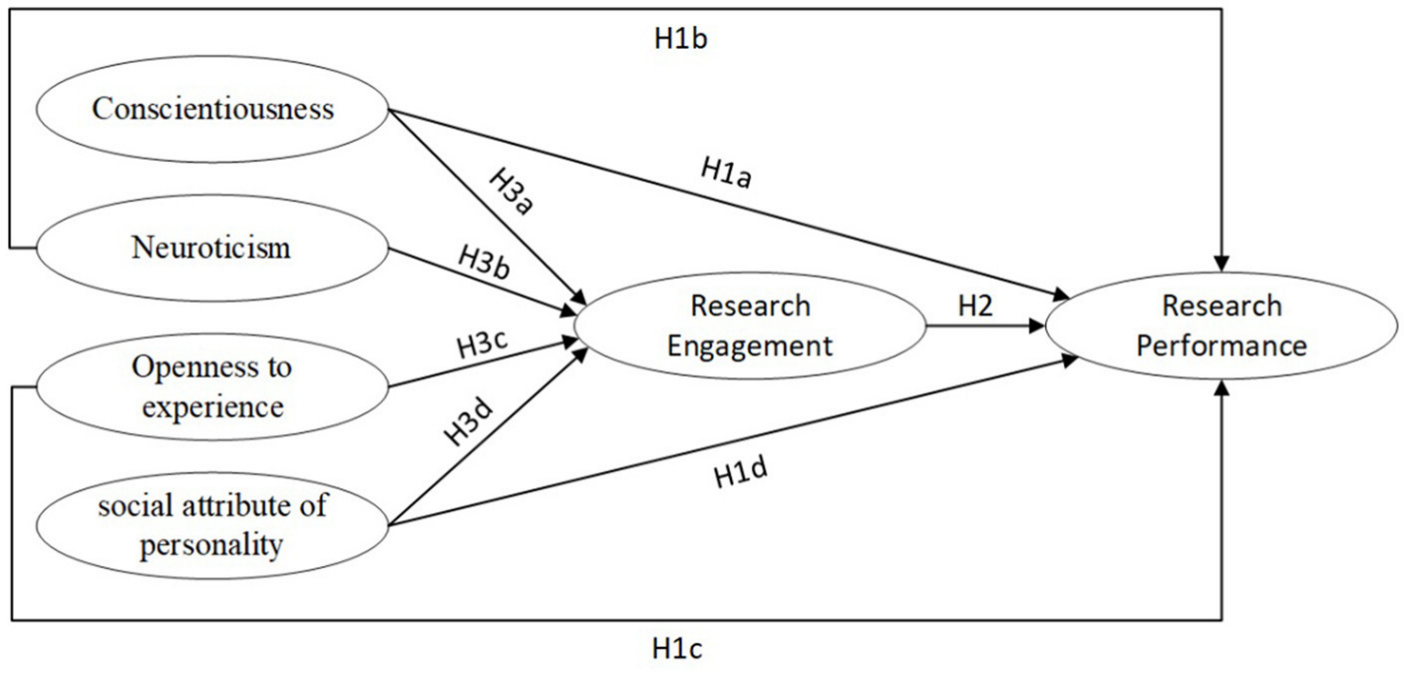
The hypothesized model. H1a/H1b/H1c/H1d denotes the total effect of conscientiousness/neuroticism/openness to experience/social attribute of personality on research performance, i.e., the sum of the direct effect of conscientiousness/neuroticism/openness to experience/social attribute of personality and the indirect effect through the path mediated by research engagement. H3a/H3b/H3c/H3d denotes the indirect effect of conscientiousness/neuroticism/openness to experience/social attributes of personality on research performance through research engagement.

The hypothesized model of this study is presented in Figure 1.

## 3 Methods

### 3.1 Participants and procedure

This study collected data from faculty members of a public university in China from May to June 2021. The majority of faculty members at this university were required to conduct research, which was important for teachers' job development, such as promotions. Thus, all the participants in this study were working as researchers.

Multiple data sources were utilized, including objective data from promotion reports and a questionnaire survey. First, to evaluate research performance, information on faculty members' publications was extracted from the university's publicly available promotion report. We adopted the weighted sum of the journal impact factor (JIF) quartile to represent research performance. Each publication of every faculty member was searched in the InCites-JCR web platform, where the JIF quartile (rank by journal impact factor) was recorded. For example, the JIF quartile of Frontiers in Psychology is Q1. The personality traits and research engagement data were collected through an online questionnaire survey. With the assistance of university department staff, questionnaires were distributed to 229 teachers, of which 211 responded. The questionnaire emphasized that all data collected are only for academic research purposes, and that we would summarize the data of all respondents for analysis rather than analyzing individuals. We guarantee the confidentiality of the information provided. All the teachers completed the questionnaire voluntarily. Finally, limited by the data on research performance we gathered, 189 questionnaires were matched and found to be valid and used as our final sample.

Among the 189 respondents, 72.5% were men and 27.5% were women. The ages of the teachers ranged from 29 to 57 years, with an average age of 39.62 years (SD = 5.432). For education, 98.4% (186) had a doctorate, and 1.6% (3) had a master's degree. Regarding tenure positions, 40.7% (77) were applying for professor or equivalent positions, while 59.3% (112) were applying for associate professor or equivalent positions.

### 3.2 Measures

We adopted established scales that have been widely used in empirical studies. All measurements were rated from 1 = strongly disagree to 7 = strongly agree. To avoid common method bias, independent variables were self-reported, while the dependent and control variables were objective criteria from the report.

#### 3.2.1 The Big Five personality traits

As the most popular personality theory in the history of personality psychology, the Big Five Model has been used to derive many measurement scales, such as the NEO-PI-R, NEO-FFI-R, IPIP-FEM, BFI, etc. (Costa and Mccrae, 1992; Goldberg, 1999; John and Srivastava, 1999; Mccrae and Costa, 2004). In this study, we adopted the Chinese version of the Mini-IPIP scale (Li et al., 2012), whose original version, the Mini-IPIP scale (Donnellan et al., 2006), was derived from the 50-item IPIP-FFM (Goldberg, 1999). The scale contains 20 items that measure extraversion, agreeableness, conscientiousness, neuroticism, and openness, using four items for each trait. The Mini-IPIP has good validity, reliability, and convergence (Donnellan et al., 2006; Li et al., 2012). The sample items for extraversion, agreeableness, conscientiousness, neuroticism, and openness are “Talk to a lot of different people at parties”, “Feel others' emotions”, “Get chores done right away”, “Get upset easily”, and “Do not have a good imagination”, respectively. In this study, extraversion and agreeableness were clustered into one factor as the social attributes of personality, so we used the items measuring extraversion and agreeableness to measure the social attributes of personality.

#### 3.2.2 Research engagement

We modified the three-item scale of work engagement used by Kraimer et al. (2019); specifically, to emphasize the research work, we used “research work” instead of “work” in the scale. Kraimer et al. (2019) tried reducing the survey length and were interested in the overall work engagement instead of the dimensions. Thus, they selected three items from the nine-item Utrecht Work Engagement Scale (UWES; Schaufeli et al., 2006). The items measuring research engagement are “At my research work, I feel full of energy,” “I am proud of the research work that I do,” and “I am immersed in my research work.” Moreover, to ensure their validity, two experienced psychologists fluent in Chinese and English translated the English versions of these scales.

#### 3.2.3 Research performance

We used publications from the faculty's promotion report to represent research performance. Considering both the quantity and quality, the research performance in this study is represented by $\overset{N}{\underset{i=1}{\sum }}Q_{i}$, where *Q*_*i*_ stands for the quality of the ith publication, and N stands for the number of publications in the teacher's representative works of their report. *Q*_*i*_ is measured by the JIF quartile and was marked 4 if the JIF quartile is Q1, 3 if Q2, 2 if Q3, 1 if Q4, and 0 if other. Considering that some publications (e.g., conference papers) without a JIF quartile also have considerable effects, such as CCFA, we dealt with them separately.

#### 3.2.4 Control variables

As mentioned in the earlier literature, demographic characteristics, such as age and gender, are strongly related to research performance (Bentley, 2011; Nielsen, 2015; Zhang et al., 2020; Fu et al., 2021; Lindahl et al., 2021). Thus, this study controlled for gender, age and the level of tenure position. In this study, gender was a dummy variable (0 = female; 1 = male), tenure positions were also a dummy variable (0 = assistant professor or equivalent positions; 1 = associate professor or equivalent positions), and age was reported in terms of years.

### 3.3 Analysis

The descriptive statistics and correlation analysis were executed by Statistical Package for the Social Sciences (SPSS) 25.0. The measurement and structural equation modeling (SEM) were conducted to test the main hypotheses by Mplus 7 software. Mplus is a SEM software package and a comprehensive set of statistical analysis tools to deal with complex models (Vandenberg, 2006). It has simple syntax, offers an accessible and simple way to test combined mediation and moderation hypotheses, and allows researchers to get indirect and total effects directly in the output file (Lau and Cheung, 2012; Sardeshmukh and Vandenberg, 2017; McLarnon and O'Neill, 2018). Thereby, it has been widely used in empirical studies (Guo et al., 2017; Han et al., 2019; Li et al., 2021).

According to the literature, the model fit would be acceptable when RMSEA is less than 0.08 and the CFI and TLA are more than 0.9 (Kline, 2005). Moreover, the bootstrapping technique was used to test the total and indirect effects (Preacher and Hayes, 2008).

## 4 Results

### 4.1 Descriptive statistics

The means, standard deviations, and correlations of all variables are presented in Table 1. The result of the correlation analysis was mostly consistent with the hypotheses. Conscientiousness was positively related to research engagement (γ = 0.215, *p* < 0.01), neuroticism was negatively related to research engagement (γ = −0.203, *p* < 0.01), openness was positively related to research engagement (γ = 0.279, *p* < 0.01), and social attributes were positively related to research engagement (γ = 0.197, *p* < 0.01). In addition, the correlation between research engagement and research performance was positive and statistically significant (γ = 0.178, *p* < 0.05). Thus, most hypotheses were supported preliminary by the results of correlation analysis.

**Table 1:** Means, standard deviations, and correlations.

**Variables**	**Mean**	**SD**	**1**	**2**	**3**	**4**	**5**	**6**	**7**	**8**
1. Age	39.62	5.432	-							
2. Gender	0.725	0.448	0.040	-						
3. Title level	0.407	0.493	0.568^**^	0.125	-					
4. Social attributes	4.653	0.767	0.105	−0.039	−0.004	(0.695)				
5. Conscientiousness	5.238	0.797	−0.041	0.028	−0.079	0.416^**^	(0.696)			
6. Neuroticism	3.675	1.016	−0.095	−0.123	−0.105	−0.111	−0.244^**^	(0.781)		
7. Openness	5.004	0.906	0.052	0.119	−0.026	0.458^**^	0.495^**^	−0.276^**^	(0.802)	
8. Research engagement	5.379	1.041	0.130	0.225^**^	0.122	0.197^**^	0.215^**^	−0.203^**^	0.279^**^	(0.859)
9. Research performance	13.57	6.317	−0.140	0.315^**^	0.097	−0.114	0.081	−0.006	0.005	0.178^*^

*N* = 189. ^*^p < 0.05 and ^**^p < 0.01; the numbers in the bracket are constructs' reliability.

### 4.2 Measurement models

The measurement models were conducted to examine the construct's convergent and discriminant validity in this study. Table 2 shows that the result of confirmatory factor analysis (CFA) indicated that the five-factor model fit the data well (χ^2^ = 114.966, df = 80, χ^2^/df = 1.437, RMSEA = 0.048, SRMR = 0.05). The loadings on each construct demonstrated good convergent validity.

**Table 2:** Results for the measurement models.

**Models**	**χ^2^**	***df***	**Δχ^2^(Δdf)**	**CFI**	**TLI**	**RMSEA**	**SRMR**
5-factor model	114.966	80		0.965	0.954	0.048	0.05
4-factor model	178.811	84	63.845 (4)^***^	0.904	0.880	0.077	0.063
3-factor model	208.174	87	93.208 (7)^***^	0.776	0.730	0.116	0.089
2-factor model	359.058	89	244.092 (9)^***^	0.727	0.678	0.127	0.097
1-factor model	582.470	90	467.504 (10)^***^	0.502	0.419	0.170	0.125

N = 189.

^***^p < 0.001.

5-factor model includes research performance and four personality traits.

4-factor model includes research performance, conscientiousness, neuroticism, and the combination of social attributes of personality and openness to experience.

3-factor model includes research performance, the combination of conscientiousness and neuroticism, and the combination of social features of personality and openness to experience.

2-factor model includes research performance and the combination of all personality traits.

1-factor model combined all factors.

Furthermore, the five-factor model fit the data better than the four-factor model combining social features of personality and openness to experience based on the five-factor model [Δχ^2^(4) = 63.845, *p* < 0.001], a three-factor model combining conscientiousness and neuroticism based on the four-factor model [Δχ^2^(7) = 93.208, *p* < 0.001], a two-factor model that combined all personality traits based on the three-factor model [Δχ^2^(9) = 244.092, *p* < 0.001], and a one-factor model that combined all factors [Δχ^2^(7) = 93.208, *p* < 0.001]. As illustrated above, the five-factor model has the highest CFI and TLI, smallest RMSEA and SRMR, and significant value of Δχ^2^(Δdf) demonstrating that the five-factor model fits the data better. Hence, the results demonstrated good discriminant validity.

### 4.3 Structural equation modeling

SEM was conducted to verify the hypothesis after examining the construct validity using the measurement model. To test the assumption that research engagement mediates the relationship between personality traits and research performance, the following two potential mediation models were compared: a full mediation model with the direct path from personality traits to research performance constrained to zero, and a partial mediation model with the unconstrained direct path. The SEM results presented in Table 3 indicated that the partial mediation model fit well (χ^2^ = 188.495, df = 134, χ^2^/df = 1.407, RMSEA = 0.046, SRMR = 0.064) and was better than the full mediation model (χ^2^ = 194.579, df = 138, χ^2^/df = 1.409, RMSEA = 0.047, SRMR = 0.065). The model that fits the data better should be selected to verify the hypotheses (Kelloway, 1998). Hence, the partial mediation model was used in this study.

**Table 3:** Comparison between partial and full mediation model.

**Models**	**χ^2^**	***df***	**Δχ^2^(Δdf)**	**CFI**	**TLI**	**RMSEA**	**SRMR**
Model 1: full mediation model	194.579	138	6.084 (4)	0.946	0.934	0.047	0.065
Model 2: partial mediation model	188.495	134		0.948	0.934	0.046	0.064

Full mediation model with the direct path from personality traits to research performance constrained to zero.

Partial mediation model with the unconstrained direct path.

### 4.4 Hypothesis testing

H1a, H1b, H1c, and H1d denote the total effect of conscientiousness, neuroticism, openness to experience, and the social attributes of personality on research performance, respectively, rather than the direct effect in the hypothesized model. For this analysis, we used point estimates and interval estimates at a 95% confidence level (*N* of bootstrapping samples = 1,000). The results of their total effects are presented in Table 4.

**Table 4:** Total/indirect effects and comparison.

	**Total effects**			**Indirect effects**		
**Paths**	**Estimates**	**95% CI**	**Accept**	**Estimates**	**95% CI**	**Accept**
(H1a) Con → RP	1.259	[0.230, 2.388]	√			
(H1b) Neu → RP	−0.187	[−0.482, −0.020]	√			
(H1c) Ope → RP	0.284	[0.002, 0.765]	√			
(H1d) Soc → RP	−2.961	[−5.726, −0.962]	√			
(H3a) Con → RE → RP				−0.012	[−0.358, 0.365]	×
(H3b) Neu → RE → RP				−0.187	[−0.482, −0.02]	√
(H3c) Ope → RE → RP				0.284	[0.002, 0.765]	√
(H3d) Soc → RE → RP				0.165	[−0.315, 0.903]	×

Con, conscientiousness; Neu, neuroticism; Ope, openness to experience; Soc, social features of personality; RE, research engagement; RP, research performance.

That zero is not in the 95% confidence interval, means the corresponding effect is significantly different from zero (p < 0.05, two-tailed).

The estimate of the total effect of conscientiousness on research performance was 1.259, and the corresponding 95% confidence interval (CI) was [0.230, 2.388]. Because zero is not in the 95% confidence interval, the total effect is indeed significantly different from zero (*p* < 0.05, two-tailed). This finding supports Hypothesis 1a.

In the same vein, the other three total effects of neuroticism, openness to experience, and the social attributes of personality on research performance were significant, supporting H1b, H1c, and H1d. Specifically, as presented in Table 4, the total effect of neuroticism on research performance was −0.187, and the corresponding 95% CI was [−0.482, −0.020]. The total effect of openness to experience on research performance was 0.284, and the corresponding 95% CI was [0.002, 0.765]. The total effect of the social attributes of personality on research performance was −2.961, and the corresponding 95% CI was [−5.726, −0.962].

**Figure 2:**
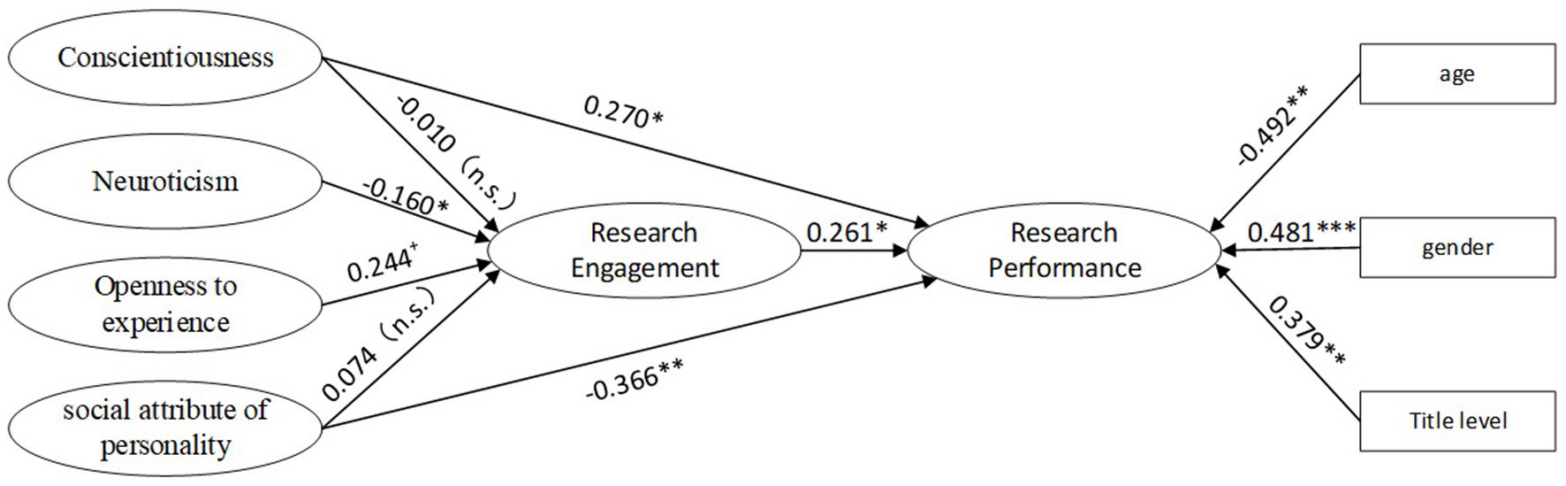
Path coefficients for the partial mediation model. The standardized estimates were reported; ^+^*p* < 0.10; *p < 0.05; **p < 0.01; ***p < 0.001.

H2 was tested by the path coefficients of the partial mediation model shown in Figure 2. H2 was supported because research engagement had a positive relationship with research performance (β = 0.261, *p* < 0.05), demonstrating that the more engagement there is, the better the performance.

As shown in Figure 2, the path coefficients from conscientiousness and the social attributes of personality to research engagement were insignificant, illustrating that H3a and H3d were not supported. The path coefficient from neuroticism to research engagement was significantly negative (β = −0.160, *p* < 0.05), and the path coefficient from openness to experience (β = 0.244, *p* = 0.055) reached the edge of a significant level (*p* < 0.05). Considering that the path coefficient from research engagement to research performance was significant, H3b and H3c received preliminary support.

To obtain further demonstrations, the indirect effects of neuroticism and openness to experience on research performance through research engagement were calculated, as shown in Table 4. The indirect effect of neuroticism on research performance through research engagement was −0.187, and the corresponding 95% CI was [−0.482, −0.020]. The indirect effect of openness to experience was 0.284, and the corresponding 95% CI was [0.002, 0.765]. Thus, H3b and H3c were verified.

We also found that the control variables significantly correlated with research performance. That is, males may have better research performance than females, and a younger age and a higher position level may lead to better performance.

## 5 Discussion

### 5.1 Theoretical implications

The Big-Five Personality theory offers a valuable framework to conceptualize personality traits and their impact on research engagement and performance. This study serves as an illustrative application of the theory within the scientific realm, an area that has received limited attention thus far. Furthermore, the study provides some theoretical implications. First, it explores the research gap in the scientific research domain of the relationship between personality traits and performance. The Big Five model has been the most popular personality theory in personality psychology. Abundant studies have focused on the relationship with its consequences (e.g., performance and turnover intention) in recent decades, but limited empirical research has focused on the relationship between personality, especially the Big Five personality traits, and research performance. After studying the earlier literature, we found that research on the antecedents of research performance generally focused on the effect of personal characteristics on demographic characteristics, such as age (Fu et al., 2021) and gender (Bentley, 2011; Nielsen, 2015; Zhang et al., 2020; Lindahl et al., 2021). Thus, our work has extended the application of the Big Five personality theory to academic domains and elucidates the impact of each personality trait on research performance.

Second, the study investigates how personalities affect research performance. This paper is probably the first to explore the role of research engagement in the relationship between personality traits and research performance, which has extended our understanding of the pathways related to personalities on research performance. Previous studies have predominantly focused on the direct relationship between personalities and research performance in the academic area (Poropat, 2009; Grosul and Feist, 2014; Djupe et al., 2020; Lindahl, 2023), and few studies have explored the inner mechanism of personalities' impact on research performance. Serrano et al. (2022) investigated the mediating role of engagement in the link between personalities and academic achievement; in particular, they focused on only two of the Big Five personality traits, conscientiousness and openness. This study considered all personality traits and found that different personality traits influence research performance in different ways. Research engagement plays a mediating role in the relationship between neuroticism/openness to experience and research performance but not in the relationship between conscientiousness/social attribute of personality and research performance.

Third, this study employs objective criteria to assess the dependent variable of research performance, which is the dependent variable in the model. Barrick mentioned that another issue of interest to many personnel psychologists is whether the objective measure of job performance results in different validity results than subjective criteria (Barrick et al., 2001). Most of the prior work on research performance has used subjective data, such as supervisors' ratings (Keller, 1997) and self-reported data (Bentley, 2011; Kim et al., 2012; Meriläinen et al., 2019), which may lead to common method bias. Kim et al. summarized 20 empirical studies on the relationship between work engagement and work performance; however, only one study used objective criteria to measure performance (Kim et al., 2012). Limited research on using objective criteria to measure performance has been conducted (Grosul and Feist, 2014; Nielsen, 2015; Ko et al., 2020; Zhang et al., 2020). Therefore, employing objective criteria may provide a more accurate reflection of actual performance compared to subjective criteria.

### 5.2 Practical implications

The implications derived from these findings offer valuable practical insights. It is imperative for university administrators to allocate greater attention to the personalities of faculty members. Universities have been continuously improving their scientific research by steps such as implementing tenure-track or talent plans. Internal pressures stemming from institutional expectations, as well as external pressures, can serve as motivational factors for faculty members. However, the internal stable traits of faculty members (e.g., personalities) must be regarded when considering the factors influencing their research engagement and performance.

To begin with, it is crucial for university administrators to consider the correlation between faculty members' personalities and research performance when devising recruitment policies, teacher training programs, personnel system reforms, and other institutional policies and initiatives. For instance, personality tests could be used as a reference in recruitment to establish the applicants' comprehensive understanding.

Second, this study has unveiled distinct associations between various personality traits and research performance, thereby offering specific implications for university administrators. For example, the findings show that faculty members with high neuroticism may experience a decline in research engagement when confronted with substantial pressure, which may provide implications that university administrators could implement measures to facilitate faculty members' research engagement, such as social support and autonomy.

### 5.3 Limitations and future research

Inevitably, this study has some limitations that could provide future research directions. First, limited by the objective criterion of research performance, the sample size was relatively small. Future empirical studies should examine a larger sample to demonstrate the relationship between personality and faculty members' research performance.

Second, other possible mechanisms underlying the effects of personalities on research engagement and performance deserve further investigation. For example, Li et al. (2021) investigated the mediating role of teaching styles in the relationship between four personality traits and work engagement.

Third, potential moderators or control variables may exist. These may strengthen, weaken, or even change the direction of the relationship. For instance, demographic and environmental factors were found to moderate the effect of four personality traits on ERP learning performance (Lea et al., 2020). Additionally, it is well-known that job performance is considerably influenced by the resources employees have in performing their tasks. Further studies ought to be conducted, taking into account the moderation effect and incorporating other pertinent control variables.

## 6 Conclusion

The current study aimed to examine the correlation between personalities and research performance while delving into the underlying mechanism through research engagement. The findings unveiled noteworthy associations between faculty members' personalities and their research performance within the university setting. Specifically, higher conscientiousness and openness to experience, as well as the lower social attributes of personality and neuroticism, may ultimately lead to better research performance. Moreover, research engagement mediated the relationship between neuroticism, openness to experience, and research performance.

## Data availability statement

The raw data supporting the conclusions of this article will be made available by the authors, without undue reservation.

## Ethics statement

Ethical review and approval was not required for the study on human participants in accordance with the local legislation and institutional requirements. Written informed consent from the patients/participants was not required to participate in this study in accordance with the national legislation and the institutional requirements.

## Author contributions

RF: Data curation, Formal analysis, Methodology, Software, Writing—original draft. YX: Conceptualization, Funding acquisition, Investigation, Methodology, Project administration, Supervision, Validation, Writing—review & editing. JW: Conceptualization, Funding acquisition, Investigation, Resources, Supervision, Writing—review & editing.
